# Bulk Magnetic Properties Arise from Micron‐Sized Supraparticle Interactions and Can be Modified on the Nanoscale

**DOI:** 10.1002/smll.202412311

**Published:** 2025-02-21

**Authors:** Andreas Wolf, Markus Heinlein, Noah Kent, Stephan Müssig, Karl Mandel

**Affiliations:** ^1^ Department of Chemistry and Pharmacy Professorship for Inorganic Chemistry Friedrich‐Alexander‐University Erlangen‐Nürnberg (FAU) Egerlandstraße 1 91058 Erlangen Germany; ^2^ Fraunhofer Institute for Silicate Research ISC Neunerplatz 2 97082 Wuerzburg Germany; ^3^ Research Laboratory of Electronics Massachusetts Institute of Technology Cambridge MA 02139 USA; ^4^ USAMcGovern Institute for Brain Research Massachusetts Institute of Technology Cambridge MA 02139 USA

**Keywords:** magnetic interactions, magnetic particle spectroscopy, matrix interactions, supraparticles

## Abstract

Magnetic supraparticles (SPs) can be employed as micron‐sized particulate additives in arbitrary objects to serve as ID‐tag or recorder of environmental triggers. Combined with magnetic particle spectroscopy (MPS), which enables read‐out of the magnetic information in ambient conditions within seconds, magnetic SPs represent a powerful approach to equip materials with information. The encoded information relies on magnetic interactions within the SPs (*intra*‐SP interactions) of chosen nanoparticles (NPs). However, possible magnetic interactions between SPs (*inter‐*SP interactions), that might alter the MPS signal as well, have been neglected so far. Herein, it is elucidated that significant inter‐SP interactions exist and that they can be tailored via adjustments in the SP structure, i.e., by defined adjustments of their *intra*‐interaction as revealed by 3D‐MuMax simulations and experiments in viscous fluids. Superparamagnetic iron oxide nanoparticle‐based SP powders with strong *inter‐*SP interactions exhibit significantly different MPS signals compared to their state after being incorporated into a matrix. Powders with weak *inter‐*SP interactions (achieved by integration of non‐magnetic SiO_2_ nanoparticles) show almost no signal change before and after incorporation. Both extremes of *inter*‐SP interactions can be beneficial for various application scenarios and can be tailored on the nano‐scale due to the interdependency of *intra*‐ and *inter*‐SP interactions.

## Graphical abstract

Magnetic supraparticles (SPs) serve as ID‐tag or recorder for environmental stimuli in objects of interest. On top of the interactions within (intra‐SP interactions), also the interactions between SPs (inter‐SP interactions) are crucial for such applications and can alter the magnetic readout. The interaction intensity on the macro‐scale can be tailored by modifying the SPs structures on the nano‐scale.

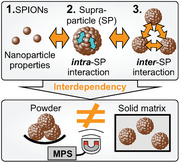

## 1 Introduction

Magnetic interaction‐based phenomena of magnetic nanoparticles, most commonly superparamagnetic iron oxide nanoparticles (SPIONs), are utilized in the fields of diagnostic biosensing,^[^ ^1^ ^]^ magnetic imaging techniques,^[^ ^2^ ^]^ and water purification.^[^ ^3^, ^4^ ^]^ Besides their intrinsic properties, namely size, shape, and crystallinity, it is the mean distance to each other that governs the magnetic interaction intensity of SPIONs.^[^ ^5^, ^6^ ^]^ An established way to control the mean distance and thereby adjust the intensity of magnetic SPION interactions is their co‐assembly with non‐magnetic nanoparticles (NPs) into micron‐sized particles – so called supraparticles (SPs).^[^ ^7^ ^]^ The magnetic interactions within SPs, herein referred to as *intra*‐SP interactions, have been exploited to create SPs with fixed magnetic fingerprints^[^ ^8^, ^9^, ^10^ ^]^ or magnetic recorder functionalities,^[^ ^11^, ^12^ ^]^ that can serve as particulate additives in arbitrary objects. A commonly used and scalable technique for producing SPs is spray‐drying.^[^ ^13^ ^]^ In this process, a dispersion containing NPs is fed into an atomizer, where a gas stream breaks it into fine droplets. Inside a heated chamber, the solvent in these droplets evaporates, causing the NPs to coalesce and form SPs.^[^ ^14^ ^]^ This concept provides the desired flexibility for precisely adjusting the size,^[^ ^15^ ^]^ shape,^[^ ^16^ ^]^ architecture,^[^ ^13^, ^17^ ^]^ and composition^[^ ^18^ ^]^ of SPs, enabling fine‐tuning of the interactions among specific nanoparticles within the SPs. Thus, the magnetic interactions of SPIONs and their magnetization response to an external field can be controlled via structural control of the SPs.

After fabrication via spray‐drying, SPs are generally in powder form, and most analytical studies have focused on examining these powders.^[^ ^14^ ^]^ However, for practical applications, incorporating SPs as particulate additives into matrices, such as bulk materials, will be necessary. Possible interactions between the SPs, that might contribute to the magnetic information, will be altered upon incorporation into bulk materials. These interactions between SPs, herein referred to as *inter‐*SP interactions, represent a yet unexplored field. The distribution of SPs within a bulk material constitutes a third critical structural hierarchy level, with the first level being the properties of the nanoparticles and the second level being the internal SP structures.

**Figure 1:**
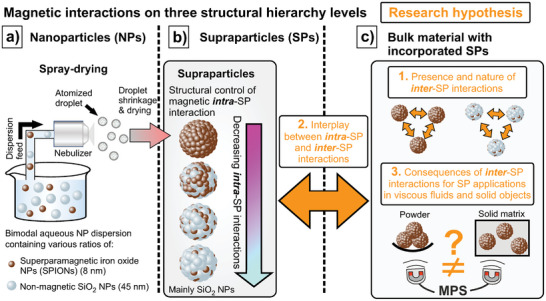
Experimental setup to probe the herein hypothesized threefold structural hierarchy with interdependent magnetic interactions. a) SPIONs as magnetic building blocks and SiO_2_ NPs as non‐magnetic building blocks are mixed in different ratios in an aqueous dispersion and spray‐dried to SPs. Note that NP building blocks are not displayed with accurate size difference (SPIONs (≈ 8 nm) and SiO_2_ NPs (≈ 45 nm)) (Figure S3, Supporting Information). b) The SP composition allows structural control of the *intra*‐SP interaction between the SPIONs, i.e., that *intra*‐SP interactions decrease with increasing share of non‐magnetic SiO_2_ NPs (highest share of SiO_2_ NPs at the bottom). c) Hypothesized interactions between SPs (*inter*‐SP interactions) constitute a third crucial level of magnetic interactions that might be interdependent with level one (a) and two (b) and bare consequences for applications of SPs in viscous fluids or solid objects and specifically their readout via MPS.

In this work, we investigate the presence of *inter‐*SP interactions and elucidate the interdependency of magnetic *intra*‐SP and *inter*‐SP interactions. For this purpose, aqueous dispersions containing varying ratios of SPIONs and non‐magnetic silica nanoparticles (SiO_2_ NPs) (**Figure** **1**a) are spray‐dried to create bimodal SPs with tailored *intra*‐SP interactions (Figure 1b). An increasing amount of SiO_2_ NPs decreases the *intra*‐SP interactions (Figure 1b).^[^ ^8^, ^12^ ^]^ Combining experimental setups of SPs being incorporated in various matrices with theoretical 3D MuMax simulations, we systematically investigate a threefold research hypothesis (Figure 1c). First, we reveal the presence of *inter‐*SP interactions and provide mechanistic insights into their nature. Second, we study the interplay of *intra*‐ and *inter*‐SP interactions, i.e., to what extent the modification of *intra*‐SP interactions changes the intensity of *inter*‐SP interactions. Third, we discuss the consequences of these findings on the application of SPs in viscous media or solid objects to serve as information‐providing particulate additive. Magnetic particle spectroscopy (MPS) was shown to be sensitive to minor changes in *intra*‐SP magnetic interactions and is the method of choice to read the information stored in magnetic SPs in ambient conditions within seconds (more details on the MPS setup and working principle can be found in the results section as well as Figures S1, S2, Supporting Information).^[^ ^8^, ^9^, ^19^ ^]^ We hypothesize, that all three structural hierarchy levels (Figure 1) are relevant for the magnetic information that can be retrieved via MPS and thus, the understanding of their contributions to the overall signal is crucial to design SPs for specific application scenarios. Based on the versatility of the spray‐drying approach and the known variety of ways to modify the *intra*‐SP interactions, the interdependency of these three structural hierarchy levels would allow to finetune the macroscopic properties of objects containing magnetic SPs by tailoring the interactions on the nano‐scale.

## 2 Results and Discussion

### 2.1 Structural Control of Magnetic Interactions Within Supraparticles (*Intra*‐SP Interactions)

SPIONs as primary magnetic signal carriers were synthesized via a well‐established co‐precipitation of iron salts in an alkaline milieu.^[^ ^4^, ^20^, ^21^ ^]^ Subsequently, they were modified with citric acid on the surface and redispersed in water to obtain a ferrofluid. The superparamagnetic behavior originates from their small diameter (≈8 nm). However, the precipitation leads to a broad size distribution and a small share of SPIONs possessed diameters of >16 nm (transmission electron micrographs and SPION size distribution: Figure S3, Supporting Information). In the size regime above 16 nm, ferrimagnetic characteristics can be present.^[^ ^22^ ^]^ Indeed, the hysteresis loop reveals a coercivity of 35 Oe (Figure S3, Supporting Information). Hence, the magnetic NPs, herein referred to as SPIONs, are strictly speaking a mix of SPIONs and ferrimagnetic iron oxide nanoparticle, whereby the share of the latter one is less than 10 wt.% (as determined from statistical transmission electron micoscopy analysis). The presence of citric acid on the SPION surface was validated via Fourier‐transformed infrared spectroscopy (FT‐IR) and thermogravimetric analysis (TGA) (Figure S4, Supporting Information). Dynamic light scattering indicated slight agglomeration of the SPIONs (D_50,number_ = 75 nm) (Figure S4, Supporting Information).

**Figure 2:**
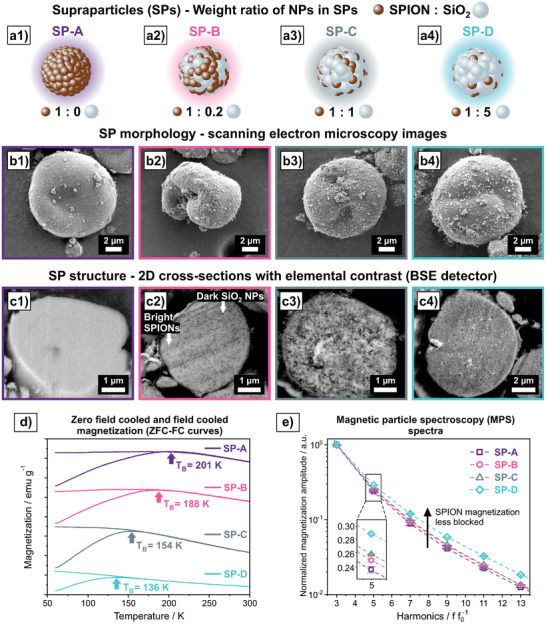
Schematic illustration of SPs containing SPIONs and an increasing share of non‐magnetic SiO_2_ NPs from SP‐A to SP‐D (weight ratio of SPIONs:SiO_2_ NPs: a1) SP‐A (1:0), a2) SP‐B (1:0.2), a3) SP‐C (1:1) and a4) SP‐D (1:5). Scanning electron micrographs of the SPs showing slightly buckled shapes and diameters of ≈10 µm for SP‐A (b1), SP‐B (b2), SP‐C (b3) and SP‐D (b4). c1–c4) Scanning electron micrographs after cross‐section polishing to display internal structure and building block distribution of SPs. The backscattered electron (BSE) detector gives elemental contrast, so that SPIONs appear brighter and SiO_2_ darker. d) Zero field cooled and field cooled (ZFC‐FC) curves. The blocking temperature (*T_B_*) can be extracted from the apex of the ZFC curve. e) MPS spectra of the SPs normalized on the third harmonic *A3* with an inset at *A5*. One MPS spectra represents the average of ten measurements. Note the logarithmic y‐scale.

To create SPs with altered *intra*‐SP interactions, the ferrofluid was mixed with colloidally dispersed SiO_2_ NPs in four distinct weight ratios and subsequently spray‐dried. The resulting SPs are referred to SP‐A (**Figure** **2**a, 1, only SPIONs), SP‐B (Figure 2a2, SPION:SiO_2_ NP weight ratio of 1:0.2), SP‐C (Figure 2a3, SPION: SiO_2_ NP weight ratio of 1:1) and SP‐D (Figure 2a4, SPION: SiO_2_ NP weight ratio of 1:5). All four types of SPs are present in the form of buckled spheres with diameters in the range of 1–10 µm (Figure 2b1–b4) (SEM overview images and SP surface analysis can be found in Figure S5 (Supporting Information), SP size distributions determined via laser diffraction can be found in Figure S7, Supporting Information). Besides size and shape, mainly the internal structure of SPs matters for the interaction of their building blocks. To guarantee SP structures in which both NP species are well‐intermixed with each other, SiO_2_ NPs with a similar size (45 nm) and surface charge as for the SPIONs were used (The results of zeta potential measurements can be found in Table S1, Supporting Information). Cross‐section polishing and subsequent scanning electron microscopy reveals the internal structure and building block distribution. The backscattered electron (BSE) detector gives elemental contrast, so that SPIONs appear brighter and SiO_2_ NPs darker (Figure 2c1–c4). SP‐A appears only bright, as there are only SPIONs in the structure (Figure 2c1). In SP‐B the SiO_2_ NPs can be observed as dark round particles, that are homogeneously distributed in the whole SP (Figure 2c2). For SP‐C, an intermixed structure of SPION clusters and SiO_2_ NPs can be seen (Figure 2c3). SP‐D predominantly consists of SiO_2_ NPs and only few SPION clusters can be found (cross‐section analysis at higher magnification can be found in Figure S6, Supporting Information).

The well‐intermixed SP structures guarantee that the average distance between SPIONs is systematically increased from SP‐A to SP‐D due to increased share of non‐magnetic SiO_2_ acting as spacing agent. Thereby, the intensity of interactions between the SPIONs in the SP (*intra*‐SP interactions) can be tailored via the weight ratio of SPIONs and SiO_2_ NPs. A possible parameter to quantify magnetic *intra*‐SP interactions is the blocking temperature (*T_B_*), which can be extracted from the apex of a zero field cooled (ZFC) magnetization curve (Figure 2d).^[^ ^23^ ^]^ *T_B_* reveals above which temperature superparamagnetic behavior is present (at a specific measurement time). Below *T_B_* SPIONs are magnetically blocked and show slow relaxation.^[^ ^22^, ^24^ ^]^ SP‐A has a *T_B_* of 201 K and*T_B_* systematically decreases with increasing share of SiO_2_ NPs down to 136 K for SP‐D (Figure 2d). Herein, we refer to these magnetically less blocked states of SPIONs as weaker *intra*‐SP interactions. Thus, a high *T_B_*, like for SP‐A, corresponds to strong *intra*‐SP interactions.

MPS is a method to investigate the interactions of magnetic colloids in ambient conditions. Throughout the last decade MPS has found increased use due to its relatively inexpensive and portable setup, as well as the ability to spectrally resolve magnetic properties of dry or liquid samples at room temperature within seconds.^[^ ^25^, ^26^ ^]^ Therefore, MPS is the instrument of choice, when it comes to the readout of magnetic SPs as information‐providing additives in bulk materials (more details on the MPS setup, working principle and data processing can be found in Figures S1, S2, Supporting Information).^[^ ^11^, ^12^, ^26^ ^]^ All four types of SPs show a gradual decay of the normalized magnetization amplitude intensities for higher harmonics, which is typical for SPIONs in MPS (Figure 2e). However, the magnetization amplitude intensities are different for all four SPs, whereby SP‐A shows the lowest and SP‐D the highest intensities for higher harmonics. Lower intensities of higher harmonics can be an indication of strong interactions and restricted relaxation of SPIONs (SP‐A).^[^ ^8^, ^11^ ^]^ This correlation between interaction intensity and the MPS signal is hypothesized to exist in these types of SPs because no dominant magnetic easy axis forms across the entire SP. Note that the logarithmic y‐scale can give the impression of non‐significant differences (Figure 2e). However, as it can be seen in the inset showing the amplitude intensity of *A5* normalized on *A3* (amplitude ratio *A5/A3*), the four spectra are distinct and represent four distinguishable interaction intensities.

In most of the previous studies that investigated magnetic SPs, they were measured in powder form and their characteristic MPS spectrum was discussed based on two factors: 1) The intrinsic properties of the magnetic signal carriers (mostly SPIONs) and 2) the interactions of the SPIONs within the SP.^[^ ^8^, ^9^, ^11^, ^12^ ^]^ Possible interactions between the SPs as a third crucial factor have been neglected so far. In the following, the existence of interactions between SPs (*inter‐*SP interactions) are confirmed and the interdependency of these three structural hierarchy levels is revealed.

### 2.2 Revealing the Existence of Magnetic Interactions Between Supraparticles (*Inter‐*SP Interactions) in MPS Fields

**Figure 3:**
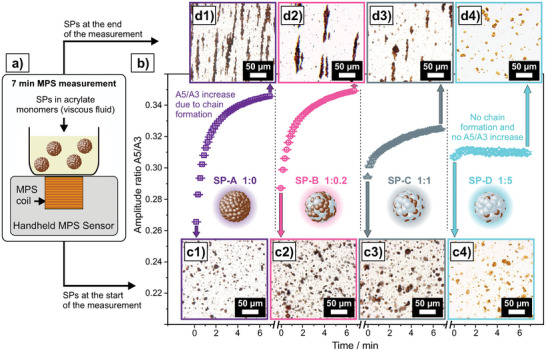
a) Schematic illustration of the measurement setup: SPs are mixed into acrylate monomers, which are present in form of a viscous fluid. The beaker containing this mixture is then placed onto a handheld MPS sensor and the measurement is run for 7‐min. b) Amplitude ratio *A5/A3* for the four different SPs (SP‐A, SP‐B, SP‐C, and SP‐D) over the course of the 7‐min MPS measurement. c1–c4) Light‐microscopy images for SPs in acrylate mixtures after UV hardening before being exposed to the MPS field. d1–d4) Light‐microscopy images for SPs in acrylate mixtures after being exposed to the MPS field for 7 min and subsequent UV hardening.

For magnetic nanoparticles, it is well‐established that their interactions enable them to form structures, such as chains, within applied magnetic fields to minimize stray fields.^[^ ^6^, ^27^, ^28^ ^]^ In the herein investigated samples, the NPs within SPs are fixed in position and cannot form structures to minimize stray fields. However, it is unknown whether the MPS field is sufficient to cause *inter‐*SP interactions that could lead to a structure formation of SPs, eventually altering their magnetic response in MPS. For this purpose, SPs were incorporated into acrylate monomers containing a photoinitiator. This system represents a viscous fluidic system, which allows SP movement. Furthermore, these samples can be hardened at any desired point in time via exposure to UV light. Plastic beakers, containing the SP‐acrylate mixture were placed on a MPS surface sensor and measured for 7 min (**Figure** **3**a). The amplitude ratio *A5/A3* in MPS over the course of these 7 min is different for the four types of SPs (Figure 3b). *A5/A3* of the first measurement is the lowest for SP‐A and the highest for SP‐D, similar to the trend in powder form. However, for SP‐A and SP‐B, a significant rise of *A5/A3* within the first few minutes of the measurement can be observed. At the end of the 7 min, *A5/A3* is about to reach a plateau. This rise is less pronounced for SP‐C and not existing for SP‐D. Light microscopy images reveal that this rise of *A5/A3* corresponds to the formation of SP chains. Before being exposed to magnetic field, all four types of SPs are randomly distributed in the acrylate matrix (Figure 3c1–c4). After being exposed to the MPS field for 7 min, chains of SPs can be observed for SP‐A, SP‐B, and SP‐C, whereby the SP chains are shorter and smaller for SP‐C (Figure 3d3) compared to SP‐A (Figure 3d1) and SP‐B (Figure 3d2). For SP‐D, no differences can be seen comparing the distribution of SPs before and after field exposure (Figure 3d4). The magnetic readout of SPs via MPS can therefore be both the cause for a structure formation over time and the method to measure the effect of such structure formation.

The degree of structure formation on the *inter‐*SP level and the corresponding signal change in MPS thus depend on the *intra*‐SP composition and thereby on the intensity of *intr*a‐SP interactions. Consequently, an interdependency of *intra*‐SP and *inter‐*SP interactions is present, whereby strong *intra*‐SP interactions cause strong *inter‐*SP interactions and vice versa. The versatility of the spray‐drying approach allows to finetune not only the SP structures and their *intra*‐SP interactions, but also the degree of *inter‐*SP interactions. To make use of this third structural hierarchy level of interactions in application scenarios, a mechanistic understanding of *inter‐*SP interactions and specifically SP chain formation is required.

### 2.3 Mechanistic Understanding of *Inter‐*SP Interactions via 3D MuMax Simulation

**Figure 4:**
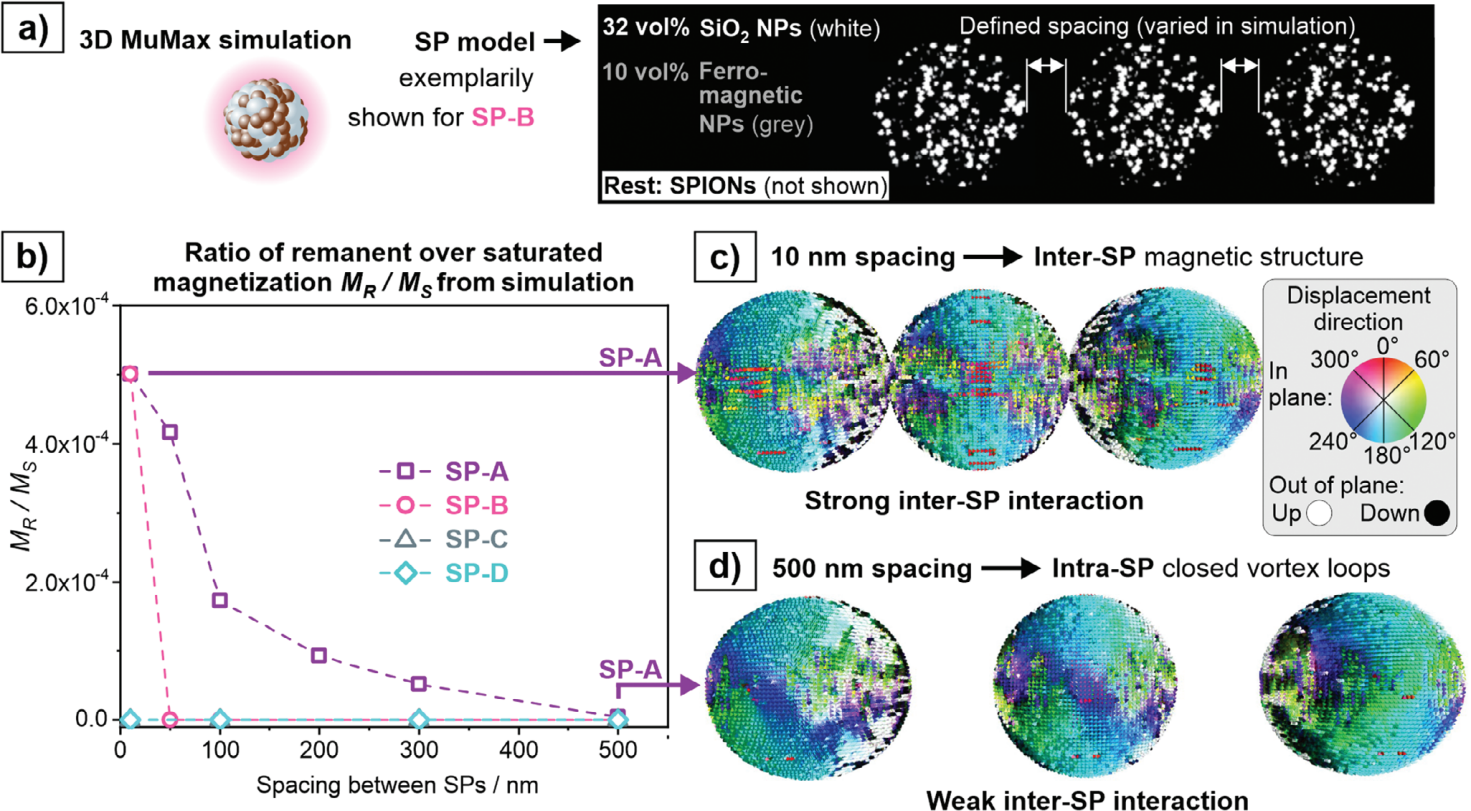
3D MuMax3 Simulations. a) Construction of a single SP in MuMax3, where a single SP is composed of millions of 10 nm diameter SPIONs with a random distribution of nonmagnetic silica (white particles) and a random distribution of 22 nm ferromagnetic magnetite particles (grey particles). b) The ratio of remanent over saturated magnetization *M_R_/M_S_* for the four types of SPs with different compositions (SP‐A, SP‐B, SP‐C, and SP‐D). When SPs with strong *intra*‐SP interaction (SP‐A and SP‐B) are closer to each other they form interparticle magnetic structures c). When they are separated or have weak *intra*‐SP interactions they form closed vortex‐like structures with no remnant field d). The color index in (c) shows the direction of magnetostrictive displacement.

The herein used MPS surface sensor employs an alternating excitation field for 50 ms, followed by 950 ms of rest. Since the SP chains build up over the course of several minutes, the 50 ms pulse is sufficient to trigger the movement of SPs toward each other, while the 950 ms rest is not long enough to fully reverse this movement due to Brownian motion in the viscous matrix. To investigate the exact nature of the physical driving force behind the chain formation, a 3D MuMax simulation was employed. For this purpose, SPs were approximated as dense spheres with a diameter of 1 µm and a cell size of 10 nm was used, so that one cell attributed to one SPION. To account for the small share of ferrimagnetic NPs, 10 vol% of the NPs were substituted with 22 nm ferrimagnetic NPs, which were randomly distributed within the SP sphere. For the mixed SPs (SP‐B, SP‐C, and SP‐D) the respective share of SiO_2_ were included into the model in the same manner (exemplarily shown for SP‐B in **Figure** **4**a). Three of these SPs were placed next to each other on a linear axis and the distance between them was systematically varied between 10 nm and 500 nm. At each distance, a simulation was run in which an external field was applied to magnetically saturate the three SPs. Then, the external field was removed and the relaxation of the three SPs in a zero‐field was simulated to obtain the ratio of remanent magnetization over saturated magnetization *M_R_/M_S_* (further details about the simulation can be found in the experimental section). The *M_R_/M_S_* ratio serves as a first‐order approximation of the particles anisotropy: magnetic systems with higher anisotropy have a higher *M_R_/M_S_* ratio.^[^ ^29^ ^]^ The higher the anisotropy of a magnetic system, the sharper the switching which results in a higher *A5/A3* ratio.^[^ ^28^ ^]^ Although this scenario used in the 3D MuMax simulation is not identical to the dynamic magnetization in MPS, it reveals the tendency of SPs to change their MPS response as a function of distance and SP composition. At distances of 500 nm no remanent magnetization is present for any of the four types of SPs. At distances closer than 500 nm a remanent magnetization occurs for SP‐A, which increases with smaller SP distances (Figure 4b). The simulation reveals, that for SP‐A the close proximity of the SPs leads to an *inter‐*SP magnetic structure, which can be referred to as strong *inter‐*SP interaction (Figure 4c). In contrast to this, the individual SPs at greater distance form closed vortex loops (Figure 4d). The remanent magnetization supports the chain formation, as the movement of the SPs toward each other has a driving force, even when the MPS excitation field is turn off. *Intra*‐ and *inter*‐SP interactions are interdependent. *Intra*‐SP interactions determine the presence and intensity of *inter*‐SP interactions. Conversely, once *inter*‐SP interactions are established, they impact the magnetic structure within the SPs, thereby altering the *intra*‐SP interactions. While the magnetic structure inside the SPs is influenced by *inter*‐SP interactions, the chemical structure is expected to remain unchanged, as the herein used SPs show no redispersion or fragmentation in water after speedmixing or exposure to magnetic fields.^[^ ^20^ ^]^

Notably, there are discrepancies between the simulation and the experimental findings. First, the remanent magnetization for SP‐B is only present at short distances and becomes zero at 50 nm, although experimentally, SP‐B behaves similar to SP‐A and attractive forces are present also at distances greater than 10 nm. Second, SP‐C shows no remanent magnetization at any distance in the simulation, even though small chains also form in case of SP‐C (Figure 3d3). These discrepancies are likely caused by clustering of SPIONs within the SPs. Although the SPIONs are distributed across the whole SP for SP‐B, SP‐C, and SP‐D, they are distributed in form of small clusters and partly web‐like structures of ellipsoidal clusters or even chains within the real SPs (Figure S6, Supporting Information). It has been experimentally shown that linear chains of SPIONS enhance magnetic anisotropy, and lead to higher *A5/A3* signal and *M_R_/M_S_*.^[^ ^28^ ^]^ The random placement of NPs within the SPs in the model does not account for these more complex structures. Furthermore, differences in sample size may contribute to this discrepancy as well (only three SPs forming a chain in the simulation compared to millions of SPs forming longer chains in the experimental sample). However, the simulation confirms that *inter‐*SP interaction are present and that the *intra*‐SP interactions determine to what extent they can occur.

### 2.4 Investigation of SP‐Chain Formation via Matrix Modifications

**Figure 5:**
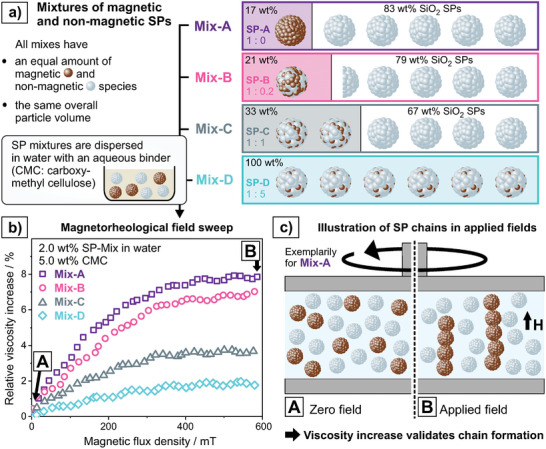
a) Schematic illustration of SPs mixtures of SP‐A, SP‐B, SP‐C, and SP‐D with non‐magnetic SPs consisting only of SiO_2_ NPs to create SP mixtures that all have the same amount of magnetic and non‐magnetic species, as well as the same overall particle volume. These SP mixtures are then dispersed in water with an aqueous binder (CMC: carboxymethyl cellulose) to obtain viscous aqueous dispersions that are suited for magnetorheological investigations. b) Magnetorheological field sweep of SP mixtures with a solid content of SPs in the dispersion of 2 wt.% and a CMC concentration of 5 wt.%. External current in the coil of the magnetorheological setup was linearly increased over the course of 2 min c) Schematic illustration of the dispersion of SP Mix‐A in the rheometer setup between the fixed bottom plate and the rotation top plate at zero field (A) and at applied field (≈600 mT) (B).

The magnetic information stored in SPs can be read out in form of amplitude ratios of higher harmonics via MPS.^[^ ^9^, ^10^, ^11^ ^]^ This information can be an ID‐tag, which should never change over time or the recording of an environmental stimulus, which should only alter the amplitude ratios in a specific scenario. For such applications, it is crucial, that the amplitude ratios do not change in an unintended way, i.e., an influencing factor that alters the amplitude ratios is overlooked and the information gets misinterpreted. To guarantee a trustworthy readout, it is crucial to understand all possible contributions to these amplitude ratios and to what extent processing of SPs, e.g. the incorporation of SP into solid objects, might alter amplitude ratios compared to the initial powder form of SPs. The findings presented in Figure 3, where the amplitude ratio *A5/A3* shows a sharp rise for SP‐A and SP‐B upon chain formation, suggest that *inter‐*SP interactions have a major contribution to the amplitude intensities in MPS, if SP chains have formed. Consequently, the MPS signal is expected to be different if chain formation is restricted and *inter‐*SP interactions are suppressed. To validate this, SPs were incorporated into matrices with increasing viscosity to gradually restrict SP movement. To guarantee similar viscoelastic properties of the samples and identical mass of magnetic species in each sample, mixtures of magnetic SPs (SP‐A, SP‐B, SP‐C, and SP‐D) and non‐magnetic SiO_2_ NPs‐containing SPs were produced (**Figure** **5**a). These SPs mixtures (Mix‐A, Mix‐B, Mix‐C, and Mix‐D) were subsequently dispersed in water with an aqueous binder, namely carboxymethyl cellulose (CMC). At first, the samples containing 5 wt.% CMC was used to perform a magnetorheological field sweep to validate the formation of SP chains in applied magnetic fields (Figure 5b). In the research field of magnetorheological fluids, this is an established method to quantify changes in viscosity due to structure formation of magnetic particles in dispersions upon exposure to external fields.^[^ ^30^ ^]^ If the herein investigated SPs show different degrees of chain formation upon field exposure, the changes in viscosity should be different for each SP mixture.^[^ ^30^, ^31^ ^]^ Indeed, the relative increase in viscosity is the highest for Mix‐A and gets systematically lower down to Mix‐D (Figure 5b). Hence, the driving force to form these SP chains is significantly greater for SP‐A compared to SP‐D, despite an equal mass of magnetic species in Mix‐A and Mix‐D. The magnetic SPs in Mix‐A first have to find each other at greater distances in the dispersion, which mainly contains non‐magnetic SiO_2_ SPs, whereas in Mix‐D each SPs contains magnetic species. However, the non‐magnetic spacer SPs do not seem to hinder the chain formation in Mix‐A (Figure 5c), whereas the *inter‐*SP interactions in Mix‐D are not sufficiently strong to form a pronounced chain network. The magnetorheological field sweep confirms the different degrees of chain formation in dependence of their *intra*‐ and *inter*‐SP interactions.

**Figure 6:**
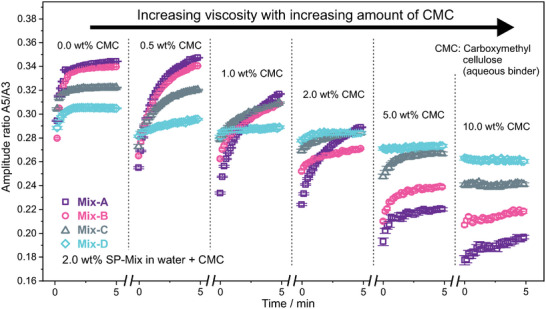
Amplitude ratio A5/A3 for SP‐mixtures in aqueous dispersions with 2 wt.% of SPs mixtures and varying CMC (carboxymethyl cellulose) concentration to systematically increase the viscosity. Each sample was measured for 5 min.

The MPS signal for SPs like SP‐A with strong *inter‐*SP interactions is expected to be dependent on the matrix viscosity, as the viscosity determines to what extent SPs can move through the matrix to form chains. In contrast, SPs like SP‐D with weak *inter‐*SP interactions should be less sensitive toward viscosity changes, since *inter‐*SP interactions and chain formation do not significantly contribute to their MPS signal. To test this, the amount of CMC was varied in the range of 0.0 to 10.0 wt.% to obtain samples with a broad range of viscosities and MPS was measured on these samples for 5 min (**Figure** **6**). At 0.0 wt.% CMC, which is SPs just in water, the amplitude ratio *A5/A3* reaches a plateau after ≈2 min. The initial rise in *A5/A3* is attributed to the combined effects of sedimentation and chain formation, with the most pronounced increase observed in SPs exhibiting strong *inter‐*SP interactions. As all SPs show similar surface charge in water, electrostatic interactions can be excluded as the main factor leading to the pronounced differences between the four types of SPs. Upon dosing CMC, viscosity increases, and as this happens, several factors influence the behavior of *A5/A3* over time. First, the initial *A5/A3* values decrease for all four types of SPs, likely due to restricted rotational freedom during their relaxation in the MPS field. However, at lower viscosities (0.5 and 1.0 wt.%), SPs with strong *inter‐*SP interactions can form chains, which significantly contribute to higher *A5/A3* values. This is evident in the upward drift of *A5/A3* for SP‐A and SP‐B, and to a lesser extent for SP‐C. As viscosity continues to rise, this effect becomes less pronounced, and the final *A5/A3* value after 5 min of measurement approaches the initial value at 0 min. In summary, it can be stated that *Inter*‐SP interactions are a driving force for SP chain formation and the corresponding rise of *A5/A3*. If chain formation is restricted, e.g. due to a highly viscous matrix, *inter*‐SP interactions are suppressed and *A5/A3* remains lower (compare Mix‐A at 0.0 wt.% CMC and 10.0 wt.% CMC in Figure 6). Vice versa, weak *intra*‐SP interactions lead to weak *inter*‐SP interactions. Since weak *inter*‐SP interactions mean little to no chain formation, *A5/A3* is less sensitive toward changes in matrix viscosity (minor difference between Mix‐D at 0.0 wt.% CMC and 10.0 wt.% CMC in Figure 6).

The herein presented mechanistic understanding of *inter‐*SP interactions bares consequences for the application scenarios of magnetic SPs that have been proposed in previous studies, namely as particulate additive in arbitrary solid objects to serve as micron sized ID‐tag or recorder for environmental triggers of interest.^[^ ^8^, ^9^, ^11^, ^12^, ^32^ ^]^

### 2.5 Consequences of *Inter‐*SP Interactions on the Application of Magnetic SPs as Particulate Additives in Solid Arbitrary Objects

**Figure 7:**
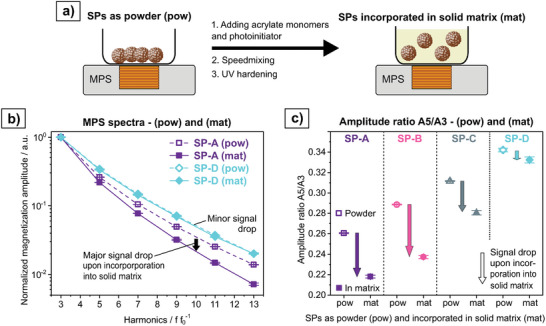
a) Schematic illustration of MPS measurement of SP powders. 10 mg of powder was measured in a plastic beaker. Subsequently acrylate monomers and a photoinitiator were added. The dispersion was mixed and UV hardened to obtain a solid polyacrylate sample. b) MPS spectra of the SPs in form of powder (pow) and incorporated into the solid matrix (mat). The spectra of SP‐A and SP‐D are exemplarily shown, as they represent the two extremes. c) Amplitude ratio *A5/A3* for SP‐A, SP‐B, SP‐C, and SP‐D for the MPS measurement on them as powders and incorporated into the solid matrix.

Assuming that the MPS signal only stems from the intrinsic properties of the magnetic signal carriers and their interactions within SPs (*intra*‐SP interactions), no signal difference between SPs as powders and SPs being incorporated into solid objects would be expected. However, this study has confirmed, that this is not the case. In fact, for SPs with strong *intra*‐SP interactions, also strong *inter‐*SP interactions are present, which can contribute largely to the MPS signal response. These *inter‐*SP interactions are expected to be altered upon incorporation of SPs into solid objects, since chain formation is restricted. To validate this, SPs as powders were measured on a MPS surface sensor. Subsequently, acrylate monomers and a photoinitiator were added, the sample mixed and UV hardened to obtain a solid sample, in which SPs are incorporated randomly in the bulk material (**Figure** **7**a). The amplitude intensities of higher harmonics of SP‐A drop significantly upon incorporation, whereas the MPS spectrum remains almost unchanged for SP‐D (Figure 7b). Also, the changes in *A5/A3* for SP‐B and SP‐C fit the previously described trend (Figure 7c). However, the comparison of MPS signals of SPs as powder and being incorporated into solid objects is intricate to generalize. The MPS signal is dependent on the extent to which rotational and/or translational movement of SPs in the sample is possible as a result of an external field, ultimately leading to a chain formation. Besides parameters like SP size and size distribution as well as shape, also environmental factors like temperature and humidity determine the flowability of powders and thus, are likely to have an impact on *A5/A3* of samples in powder form. The herein investigated powders were stored in ambient conditions. Powders being further dried and stored differently, e.g. in an inert atmosphere, might show different values for *A5/A3*.

A general statement that can be derived from these findings is that the stronger the *intra*‐SP interaction are, the stronger are also the *inter‐*SP interactions and the higher is the potential contribution of *inter‐*SP interactions to the value of *A5/A3*. If the *inter‐*SP interactions are suppressed, e.g. due to incorporation into a solid object, *A5/A3* is significantly lower compared to an environment in which the *inter‐*SP interaction can take place, e.g. in powders or fluids with low viscosity.

## 3 Conclusion

We successfully fabricated four types of SPs with distinct magnetic interactions by co‐spray‐drying SPIONs and SiO_2_ NPs with similar hydrodynamic size and surface charge in different weight ratios. The higher the share of non‐magnetic building blocks is, the lower is the blocking temperature *T_B_* and the higher are the amplitude intensities of higher harmonics in MPS. The intensity of *intra*‐SP interactions can be adjusted on a continuous spectrum by modifying the SP architecture based on the versatility of the spray‐drying approach.

**Figure 8:**
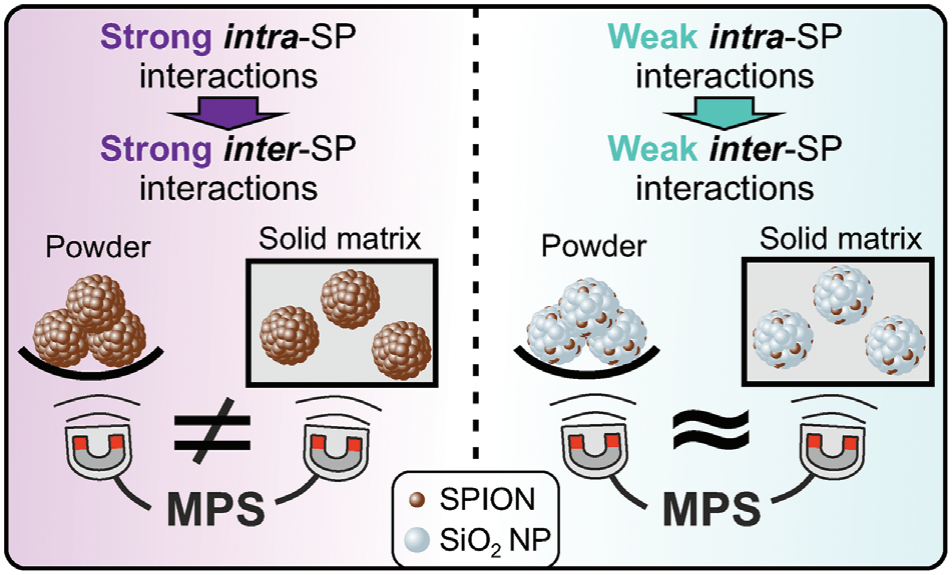
Consequences of inter‐SP interactions for applications of SPs as particulate additives in arbitrary solid objects in a nutshell: Strong inter‐SP interactions lead to significant MPS signal differences comparing powder and SP incorporated into the object of interest, whereas SPs with weak inter‐SP interactions show minor signal differences.

We revealed the existence of magnetic interactions between SPs (*inter‐*SP interactions) empirically and cross‐validated these findings via 3D MuMax simulation. The *inter‐*SP interactions represent a driving force for SPs to form chain‐like structures in applied fields, which significantly alter the MPS signal. The MPS signal for SPs with strong inter‐SP interactions differs significantly depending on the environment (**Figure** **8**). It varies between conditions where SPs can form chains, such as in powders or low‐viscosity fluids, and those where they are part of solid bulk objects or highly viscous fluids. The intensity of *inter‐*SP interaction depends on the *intra*‐SP interactions. Consequently, *inter‐*SP interactions can be tailored by adjusting the SP architecture. Multiple structural hierarchy levels, ranging from the SPION properties on the nanometer‐scale to the sample properties on the millimeter‐scale matter for the MPS signal and are in fact interdependent. This interdependency allows to adjust the macroscopic properties of objects containing magnetic SPs, by tailoring the internal SPs structure on the nanometer scale.

These findings represent both, challenges and opportunities regarding the design of magnetic SPs as smart particulate additives. The multitude of influencing parameters on the MPS signal can make trustworthy MPS data interpretation more intricate. However, this variety of influential environmental parameters also creates the opportunity to sense or record such properties, expanding the playground of magnetic SPs even further. SPs with strong *inter‐*SP interaction could for instance be designed to sense viscosity changes or to record the internal softening of bulk materials or in perspective many more other changes in the matrix of the material in which the particles are embedded in. Especially in closed environments like damping systems, MPS could provide a non‐destructive measurement of viscoelastic properties from the outside.

## 4 Experimental Section

### Materials and Synthesis—Chemicals

Iron (III) chloride hexahydrate (FeCl_3_·6 H_2_O, >99%) and iron (II) chloride tetrahydrate (FeCl_2_·4 H_2_O, >99%) and ammonia (NH_3_ (aq.), 25%) were received from Carl Roth. Citric acid monohydrate (C_6_H_8_O_7_∙H_2_O, CA, ACS grade) was bought from PanReac AppliChem. Acrylate monomers were purchased from Arkema (trimethylpropane triacrylate (TMPTA): SR351; ethoxylated TMPTA (TMPTA‐E): SR9035). The photoinitiator IRGACURE 184 was used. Sodium carboxymethylcellulose (CMC; degree of substitution: 1.2) was bought from Sigma Aldrich. Silica nanoparticles (SiO_2_ NPs) were provided by Chemiewerk Bad Köstriz GmbH. All chemicals were used without further purification. For all synthesis procedures, deionized water and technical ethanol were used.

### Materials and Synthesis—SPION Synthesis

Iron oxide nanoparticles were synthesized using a well‐established co‐precipitation method in alkaline milieu, as previously reported in the literature.^[^ ^4^, ^11^, ^20^, ^21^ ^]^ FeCl_3_·6·H_2_O (10.80 g, 40 mmol) and FeCl_2_·4·H_2_O (3.98 g, 20 mmol) were dissolved in 125 mL of deionized water and stirred at room temperature in a beaker. In a second beaker, 180 mL of a 5 wt.% ammonia solution was prepared. Both solutions were pumped (peristaltic pump, Ismatec MCP, flow rate 500 mL min^−1^) into a static mixer (plastic spiral bell mixer 7700924, Nordson Deutschland GmbH) and collected in a third beaker. Each solution had a flow rate of 500 mL min^−1^. The tube carrying the ammonia solution to the mixer was shorter, ensuring the ammonia reached the mixer 1 s before the iron salt solution. The resulting black precipitate, was stirred for an additional 2 min, then magnetically separated and washed three times with 250 mL of deionized water. A permanent magnet (Neodym, N40) was placed under the beaker for magnetic separation. For surface capping, the SPIONs were magnetically separated again and 500 mL of 0.05 m citric acid (CA) was added. After stirring for 5 min, the SPIONs were magnetically separated and washed four times with 200 mL of ethanol. The SPIONs were then redispersed in water. The resulting ferrofluid was sonicated for 5 min (Branson Ultrasonic Sonifier, output: 20, duty cycle 100%) and afterward adjusted to pH 9 via the addition of ammonia.

### Materials and Synthesis—Supraparticle Synthesis

The NP dispersions for spray‐drying were prepared by mixing the desired amounts of SPION and SiO_2_ NP dispersions with deionized water while keeping the solid content at 5 wt.%. Weight ratios (SPIONs: SiO_2_ NPs) of 1:0, 1:0.2, 1:1, 1:5 and 0:1 were mixed. SPs were fabricated at the spray‐dryer B290 from BÜCHI Labortechnik GmbH. Compressed air was used for spraying and drying. The spray gas was set to 473 L h^−1^ and the aspirator was kept at 85%. The inlet temperature was set at 120 °C, and the pump rate was fixed at 20 % (7 mL min^−1^).

### Materials and Synthesis—SP Incorporation into Acrylates

For the incorporation of SPs in acrylates 0.40 g of TMPTA, 1.60 g of TMPTA‐E and 0.04 g of IRGACURE 184 were mixed 12 mL plastic beaker using a speedmixer (Hausschild) operated at 3000 rpm for 2 min. The mixture rested for 4 h. Then, 0.04 g of the SPs were added and mixed again (3000 rpm, 2 min). For performing microscopy, a droplet of the acrylate mixture containing SPs, was applied on thin glass microscopy slides and a second slide was placed on top of the droplet, so that the sample spread out as thin film between the slides. These samples were then treated with UV light for 2 min (Bluepoint 3, Dr. Hönle AG) to obtain the samples without magnetic field exposure (Figure 3c). Further samples were placed next to the MPS surface sensor for 7 min before turning on the UV light (Figure 3d). As reference, another set of samples was prepared, which were placed 5 cm next to a permanent ferromagnet magnet (Q‐51‐51‐25‐N, supermagnete.de) for 10 s, before turning on the UV light for 2 min. The permanent magnet remained next to the sample during the UV‐hardening.

### Materials and Synthesis—SP Incorporation into Viscous Aqueous Matrices

At first magnetic SPs (SP‐A, SP‐B, SP‐C, and SP‐D) were mixed with non‐magnetic SiO_2_ SPs to create SP mixtures with equal amounts of magnetic and non‐magnetic species (see Figure 5a for details on the ratios). 2 mL of deionized water was filled into 12 mL plastic beakers and the respective amount of carboxymethyl cellulose (CMC) was added to achieve mixtures of 0.0, 0.5, 1.0, 2.0, 5.0, and 10.0 wt,% CMC in water. Then, 0.04 g of a SP mix was added and mixed with a speedmixer at 3000 rpm for 3 min. The mixture rested for 24 h and was then mixed again at 3000 rpm for 2 min. Subsequently, the beaker was placed in the MPS surface sensor for MPS measurements (Figure 6).

### Characterization Methods—Magnetic Particle Spectroscopy (MPS)

MPS measurements were performed with two different MPS sensors, referred to as static MPS sensor and surface sensor, both being connected to a MPS control unit (PureDevices GmbH). The static MPS sensor was used for all powder samples, operating with a sinusoidal alternating magnetic field of 20 kHz in a range of ± 23.87 kA m^−1^ (30 mT) at 37 °C. The spectra were background corrected with a reference measurement without a magnetic sample. For each powder measurement several milligrams of powder were filled into a cylindrical glass vial. Three different vials were prepared for each powder sample and each vial was measured ten times. Displayed data of the amplitude ratio *A5/A3* were the mean average values of these three samples. A full MPS spectrum that was displayed (Figure 2e) was derived from one representative sample out of the three equally prepared sample vials. The MPS surface sensor operated at 20.05 kHz in a range of ± 15.92 kA m^−1^ (20 mT) at ambient conditions. For measurements with the surface sensor 25 mL plastic beakers containing the samples were placed on top of the sensor centered above the coil. Ten individual measurements were averaged for each data point. The surface sensor was used for all MPS data displayed in Figures 3, 6, and 7.

### Characterization Methods—Scanning Electron Microscopy (SEM)

SEM was performed with a JSM‐F100 (JEOL) at a working distance of ≈ 6 mm applying an acceleration voltage of 2 kV (field emission). For morphological analysis using secondary electron detection, SP powders were spread on carbon pads (Plano). Back‐scattered electron detection was used for elemental contrast mode analysis applying an acceleration voltage of 8 kV.

### Characterization Methods—Cross‐Section Preparation of Supraparticles

For cross‐section analysis, supraparticle powder was attached to a conductive carbon pad and sandwiched between two silicon wafers. Cross‐sections were cut using an IB‐19530CP (JEOL) with an Argon (Ar) plasma beam for 10 h in pulse mode (40 s on, 20 s off). The Ar flow rate was set to 5.3 sccm, and the accelerating voltage to 8 kV.

*Hysteresis loops* were performed with a superconducting quantum interference device (SQUID) MPMS3 (Quantum Design Inc.) at 300 K. The magnetic field range was set to ±30 000 Oe, the measurement speed to 5 Oe s^−1^ between ±5000 Oe and to 50 Oe s^−1^ beyond.

*Zero field cooled* (ZFC) and *field cooled* (FC) measurements were performed with a vibrating sample magnetometer (VSM, VersaLabTM 3T, Cryogenfree Vibrating Sample Magnetometer). The samples were demagnetized at 293 K by setting an initial field of 10 kOe and decreasing the field stepwise to zero by oscillating at 200 Oe s^−1^. Samples were cooled down to 60 K via a cryocooler‐based cooling system at zero field. Then an external field of 10 Oe was applied and the samples were heated to 300 K at  K min^−1^ and again cooled down to 60 K at 1 K min^−1^ in the 10 Oe field. The magnetization M was measured by vibrating the samples at 40 Hz. One data point was delivered for M measured within 1 s (averaging time).

### Characterization Methods—Transmission Electron Microscopy (TEM)

TEM images were taken using a LEO912 Omega (Zeiss) applying an acceleration voltage of 80 kV. Samples were prepared from diluted ethanolic sample dispersions to carbon‐coated copper grids (Plano).

### Characterization Methods—X‐Ray Diffractometry (XRD)

XRD was performed with a D8 Advance diffractometer (Bruker) equipped with a Lynxeye XE‐T detector employing Cu Kα radiation.

*Magnetorheological measurements* were conducted with a Dynamic Mechanical Analyser EC‐Twist 502 (Anton Paar) equipped with a MRD70 unit and a PP20/MRD measurement head. A PS‐MRD current source was used to apply the magnetic field and a magnetometer FF54 (Magnet‐Physik Dr. Steingroever) was used to measure the magnetic field. For each sample, 0.31 mL was used. At first, the shear rate was increased from 0.1 to 10 ^−1^ within 100 s and held constant at 10 s^−1^. Then the current was linearly increased from 0 to 5 A with 0.1 A steps and a step time of 3 s. Concurrently the resulting magnetic field was measured.

*Light Microscopy* was performed with an Olympus BX53 M, equipped with a SC50 camera.

### Characterization Methods—Dynamic Light Scattering (DLS)

DLS was performed with an Anton Paar Litesizer500 at 25 °C. The sample was further diluted with a pH 9 stock solution (deionized water and ammonia) to reach a transmittance of ≈ 75%. One measurement contained six individual runs.

### Characterization Methods—Zeta Potential

Zeta potential measurements were performed with a Zetasizer Nano ZS from Malvern Instruments. NPs were measured in form of a diluted dispersion at pH 9 (ammonia adjusted) and SP were measured as aqueous dispersion at pH 7. Each sample was measured five times.

*Laser diffraction* (Fraunhofer diffraction, Microtrac S3500 Model Bluewave M3551‐1W‐BU00‐3000‐0000‐000‐2 M from Microtrac with Liquid Dispenser model DIF2022; Malvern Instruments Limited) was utilized to determine the size distribution of supraparticles.

### Characterization Methods—Fourier‐Transform Infrared Spectroscopy (FT‐IR)

FT‐IR was performed on an iS5 spectrometer (Nicolet) with an attenuated total reflection setup. The spectra were averaged from 32 individual measurement runs. Spectra were recorded in the range of 400–4000 cm^−1^ with a resolution set to 2.0 cm^−1^ and a step width of 0.241 cm^−1^. The software OMNIC (Thermo Scientific) was used for data collection.

### Characterization Methods—Thermogravimetric Analysis (TGA)

TGA was carried out with a TG 209 F1 Libra (Netzsch). The material was heated in synthetic air from 30 to 1000 °C with a heating rate of 10 K min^−1^ and a gas flow of 50 mL min^−1^.

### Simulation

MuMax3 simulations were performed with a 10 × 10 × 10 nm cell size. Three 1 µm diameter spheres were initialized with magnetite magnetic properties, but with no exchange interaction. This allows each cell to act as a 10 nm magnetite SPION. Based on microscopy images, 10 % of the magnetite particles were estimated to be sufficiently large to be ferrimagnetic and accounted for in the simulation as 22 nm ferrimagnetic nanoparticles. The ferrimagnetic magnetite nanoparticles were randomly distributed within the magnetite of the SPs, with ferrimagnetic exchange turned on for the ferrimagnetic nanoparticles. For SPs with silica inclusions, 45 nm non‐magnetic spacer particles were randomly distributed within the SP. For SP‐D, ferrimagnetic and superparamagnetic magnetite were randomly distributed within a non‐magnetic silica superparticle; this was done as the majority of SP‐D was silica.

## Conflict of Interest

The authors declare no conflict of interest.

## Author Contributions

A.W. contributed in conceptualization, investigation, methodology, data curation, funding acquisition, supervision, validation, visualization, and writing—original draft and review and editing. M.H. contributed in conceptualization, investigation, methodology, data curation and review and editing. N.K. contributed in investigation, methodology, simulation, and writing—review and editing. S.M. contributed in conceptualization, supervision, visualization, and writing—review and editing. K.M. contributed in conceptualization, funding acquisition, project administration, resources, supervision, and writing—review and editing.

## Supporting information



Supporting Informations

## Data Availability

The data that support the findings of this study are openly available in Zenodo at https://doi.org/10.5281/zenodo.14280748, reference number 14280748.
