# Supplementary material for: Bulk Magnetic Properties Arise from Micron‐Sized Supraparticle Interactions and Can be Modified on the Nanoscale
Source: Small. 2025 Feb 21;21(13):2412311. doi: 10.1002/smll.202412311 (PMC11962683; doi:10.1002/smll.202412311)
Supplement: Supplementary file 1 — Supporting Informations [file SMLL-21-2412311-s001.docx]

Supporting Information

Bulk magnetic properties arise from micron-sized supraparticle interactions and can be modified on the nanoscale

Andreas Wolf, Markus Heinlein, Noah Kent, Stephan Müssig and Karl Mandel*

**Magnetic particle spectroscopy (MPS): Instrument setup, working principle and data processing**

MPS measurements were performed with two different setups in this study. The MPS spectra of SP powders presented in Figure 2e was measured with an established MPS device from Pure Devices (see Experimental section for details) (Figure S1a). In this setup, the isotropic magnetic field within the coil is considered the measurement volume (Figure S1b). All other MPS measurements within this study were performed with a custom-built handheld surface sensor (Figure S1c). This sensor is designed to utilize the anisotropic magnetic field outside of the coil (Figure S1d). With the measurement volume outside of the coil, measuring the bulk volume ob arbitrary objects is possible. Herein, the plastic beakers containing either SP powder, liquid or hardened acrylates, as well as aqueous CMC mixtures were placed on top of the surface sensor and centered in the middle of the coil.


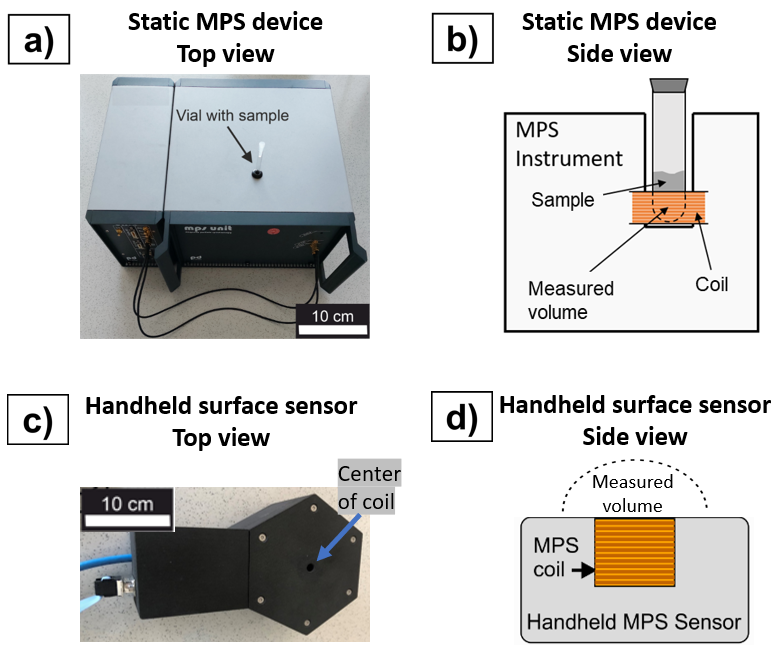


**Figure S1.** a) Picture of established static MPS device from top view. b) Scheme of static MPS device from side view. c) Picture of MPS surface sensor from top view. d) Scheme of MPS surface sensor from side view.

In magnetic particle spectroscopy (MPS), a sinusoidal alternating magnetic field (Figure S2a) is applied to induce magnetization within the sample. The sample’s magnetic properties (e.g., dia-, para-, ferro-, or superparamagnetic behavior) influence its magnetic response. For ferro-/ferri- and superparamagnetic samples, which exhibit non-linear magnetization behavior (Figure S2b), the temporal magnetization deviates from the sinusoidal shape of the excitation field (Figure S2c).

The interaction of the alternating magnetic field with the sample generates an induced voltage in the pick-up coils of the measurement device (Figure S2d). This overall induced voltage (U_i_​) combines the excitation field's contribution (U_e_​) (Figure S2e) and the sample's magnetic response (U_p_​) (Figure S2f). By applying a Fourier transformation to the induced voltage, a harmonic spectrum is obtained (Figure S2g), showing the frequency of the excitation field and its higher-order harmonics that are caused by the non-linear magnetization of the sample. Subtracting the transformed excitation field signal (Figure S2h) isolates the sample's unique harmonic spectrum (Figure S2i).

The intensities of the higher harmonics depend on the parameters of the externally applied field, the sample mass, the intrinsic magnetic properties of the sample, and any interactions within the sample. The absolute values of the amplitude intensities can be different for different MPS devices like the two herein used sensor geometries. Herein, this is due to geometrical effects as well as field strength. (20 mT for the surface sensor and 30 mT for the static MPS device). Normalization on lower harmonic amplitudes (most commonly A3) is an established method to create comparability of samples with varying sample mass (Figure S2j and l).^[1,2,3]^


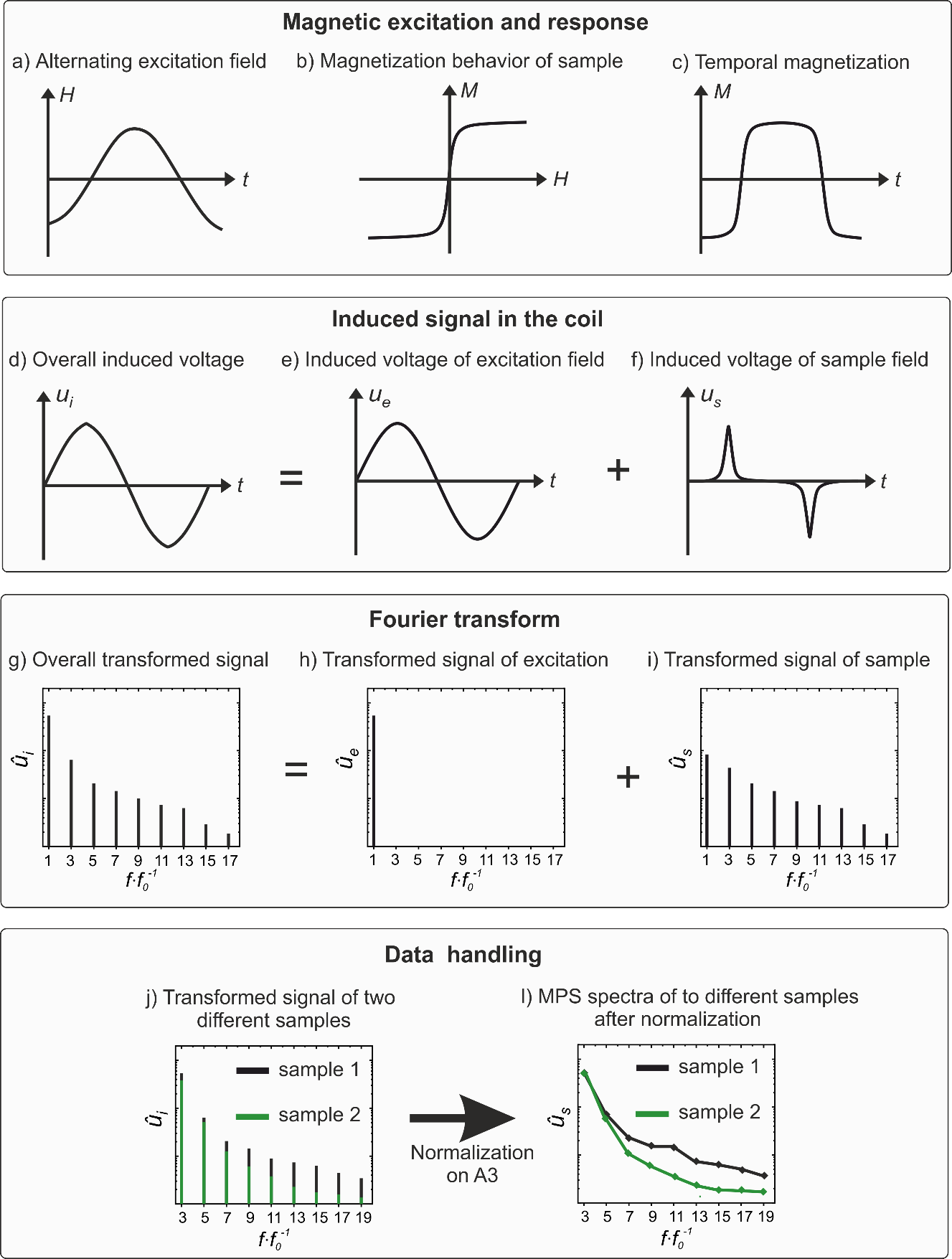


**Figure S2.** Schematic representation of the measurement principle and data processing for MPS. a) A sinusoidal magnetic field is applied. The sample's magnetization behavior (b) influences the overall temporal magnetization (c). The total induced voltage (d) comprises contributions from both the excitation field (e) and the sample (f). Applying a Fourier transformation to the signal generates a frequency spectrum (g). Subtracting the transformed signal of the excitation field (h) isolates the sample's spectrum (i). (j) To account for differences in sample mass, higher harmonics are normalized to lower harmonics, yielding characteristic amplitude ratios (l).

**Characterization of superparamagnetic iron oxide nanoparticles (SPIONs)**


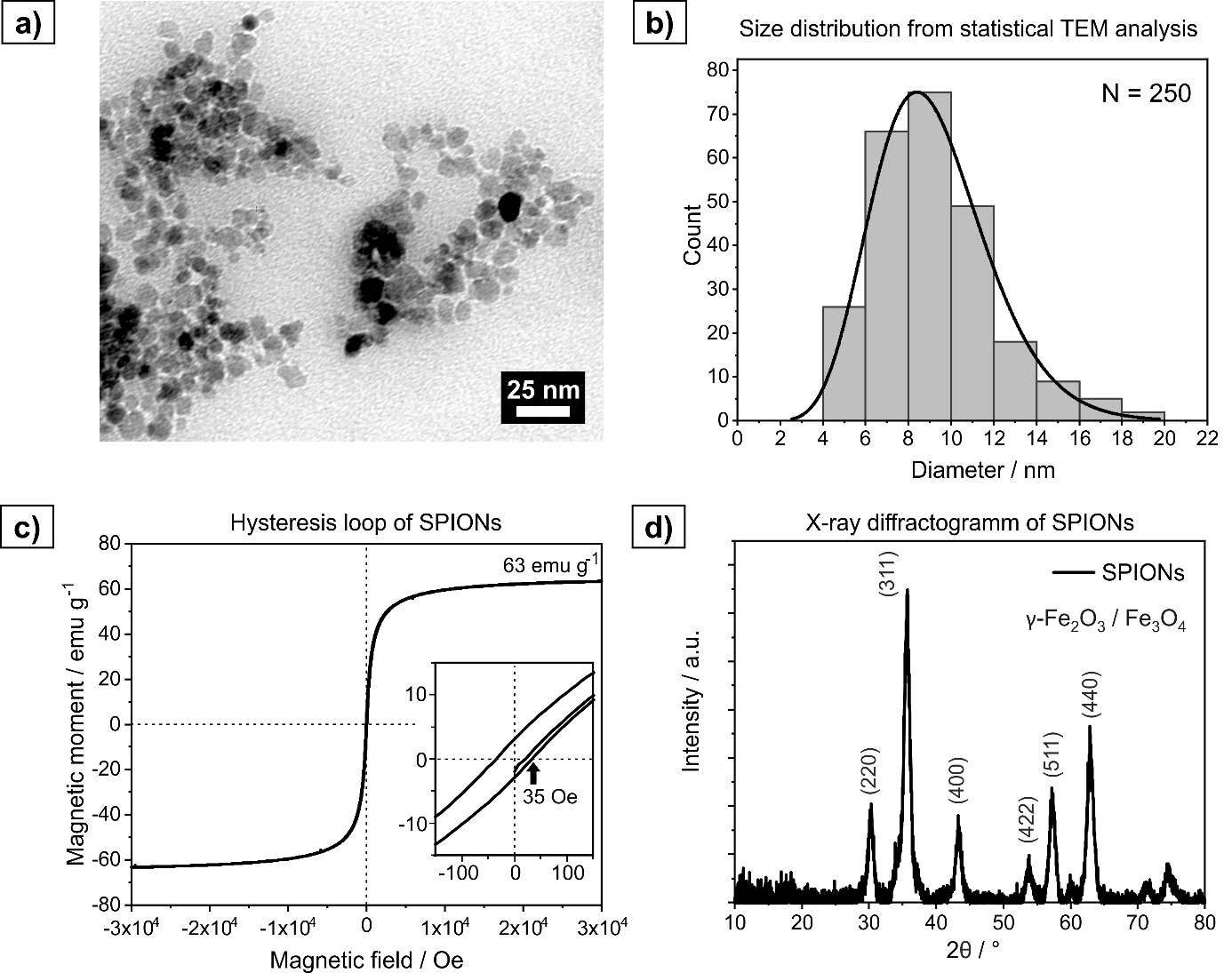


**Figure S3.** Characterization of superparamagnetic iron oxide nanoparticles (SPIONs). a) Transmission electron microscopy image of SPIONs. b) Size distribution histogram of SPIONs determined via statistical analysis of TEM images (Gamma distribution maximum at 8 nm). c) Hysteresis loop of SPIONs exhibiting a saturation magnetization of 63 emu g^-1^ and a coercivity of 35 Oe. d) X-ray diffractogram of SPIONs indicating the presence of magnetite (Fe_3_O_4_) and/ or maghemite (y-Fe_2_O_3_). Note that, the exact share of Fe_3_O_4_ and γ-Fe_2_O_3_ is not of importance for the herein discussed magnetic interactions, as they both possess similar magnetic properties.


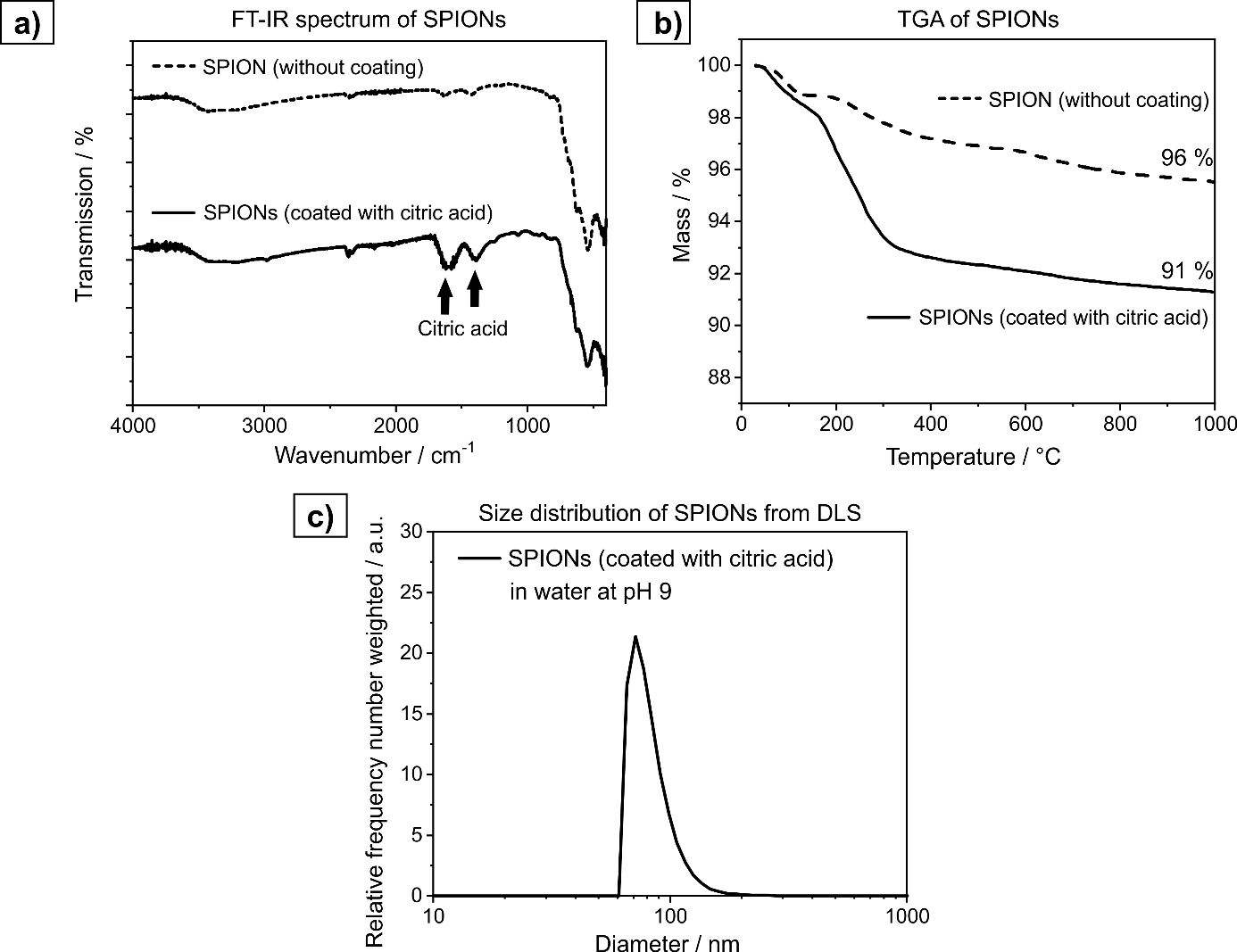


**Figure S4.** a) Verification of the presence of citric acid via Fourier-transform infrared spectroscopy (FT-IR). b) Thermogravimetric analysis (TGA) indicating the presence of citric acid by a total weight loss of 9 % compared to the non-functionalized bare SPIONs with a weight loss of only 4 %. c) Number-weighted size distribution measured via dynamic light scattering (DLS) indicating slight agglomeration of SPIONs at pH 9.

**Characterization of of supraparticles**


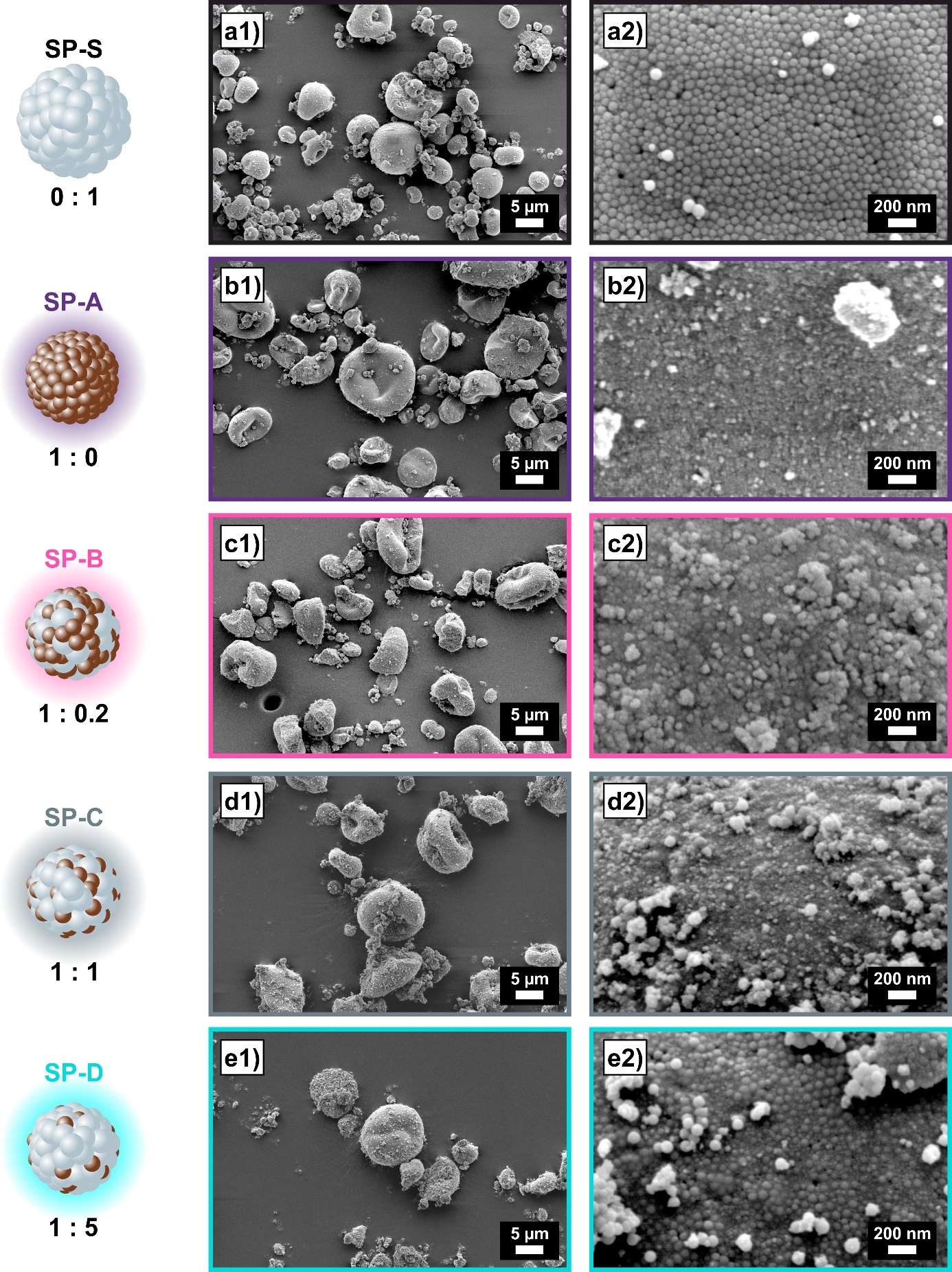


Figure S5. Scanning electron micrographs of supraparticles. Overview images and surface analysis for SP-S (only composed of SiO_2_ NPs) (a1 and a2), SP-A (only composed of SPIONs) (b1 and b2), SP-B (c1 and c2), SP-C (d1 and d2) and SP-D (e1 and e2).


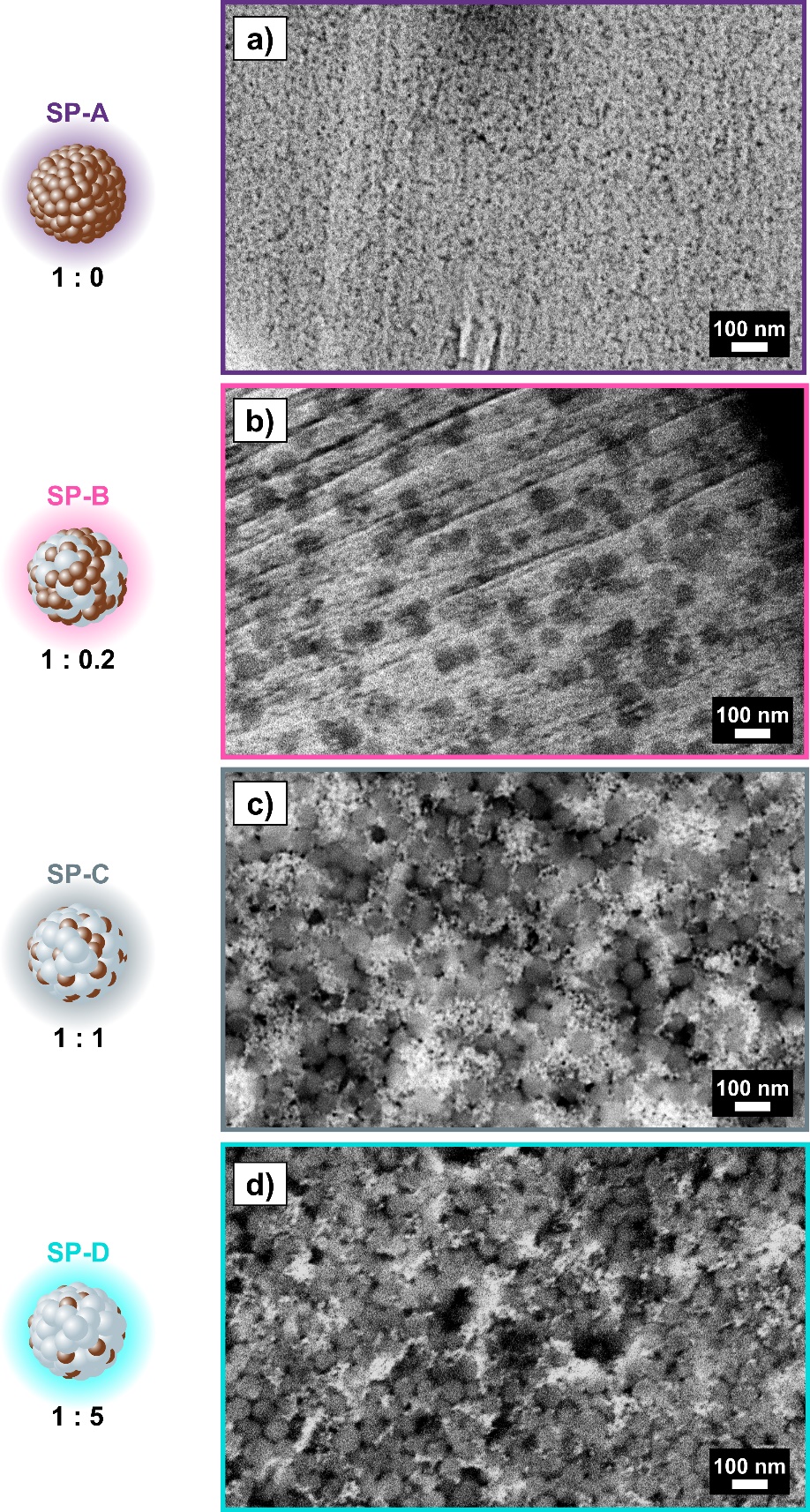


Figure S6. Cross-section analysis of supraprticles. Scanning electron micrographs for which the backscattered electron detector was used to create an elemental contrast: SPIONs appear bright and SiO_2_ NPs appear darker. a) For SP-A only bright SPIONs can be observed. b) The small share of SiO_2_ NPs of SP-B can be found as dark spherical NPs within the SP, which mainly consists out of SPIONs. The SiO_2_ NPs are randomly distributed within the SP. c) SP-C, which consists of equal amounts of SPIONs and SiO_2_ NPs by weight, has an intermixed structure of both species. The same counts for SP-D, but with a lower share of SPIONs.


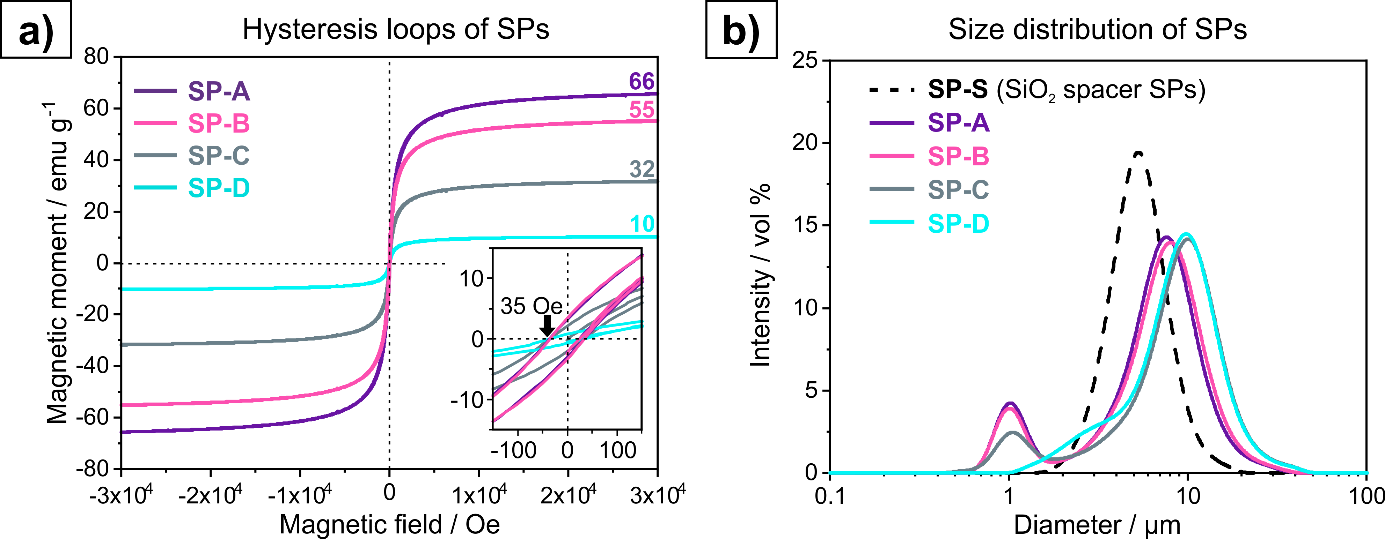


Figure S7. a) Hysteresis loops of SPs with saturation magnetizations that correspond well to the weight share of SPIONs in the SPs. SP-A: Pure SPIONs and therefore a similar saturation magnetization than the SPIONs. SP-B: A weight share of 83.3 % of SPIONs and thus, a slightly reduced saturation magnetization. SP-C: A SPION weight share of 50 % resulting in roughly half of the saturation magnetization of SP-A. SP-D: A SPION weight share of 16.7 % leads to roughly 1/6 of the saturation magnetization of SP-A. All SPs show a coercivity of about 35 Oe, indicating a small share of ferrimagnetic iron oxide nanoparticles. b) Volume weighted size distribution of SPs. All SPs possess a similar size distribution with sizes in the range of several micrometers. SP-A, SP-B and SP-C also have a smaller fraction of SPs with diameters around 1 µm.

**Table S1**. Zeta potential measurements of NPs and SPs. NPs (SPIONs and SiO_2_ NPs in form of KS4550) were measured as prepared for spray-drying in form of an aqueous dispersion at pH 9 and for zeta potential measurements further diluted with a pH 9 stock solution. SPs were dispersed in de-ionized water at pH 7. The values presented in the table are average values of five individual measurements.

| **Sample** | SPIONs | KS4550 | SP-A | SP-B | SP-C | SP-D |
| --- | --- | --- | --- | --- | --- | --- |
| **pH** | 9 | 9 | 7 | 7 | 7 | 7 |
| **Zeta**  **potential**  **/ mV** | - 40.1±1.0 | - 48.0±0.9 | - 32.0±0.7 | - 31.0±1.0 | - 33.2±0.3 | - 36.3±0.3 |

References

[1] S. Biederer, T. Knopp, T. F. Sattel, K. Lüdtke-Buzug, B. Gleich, J. Weizenecker, J. Borgert, T. M. Buzug, *J. Phys. D: Appl. Phys.* **2009**, *42*, 205007.

[2] S. Müssig, F. Fidler, D. Haddad, K.‐H. Hiller, S. Wintzheimer, K. Mandel, *Adv Mater. Technol.* **2019**, *4*, 877.

[3] A. Wolf, J. Sauer, K. Hurle, S. Müssig, K. Mandel, *Adv. Func. Mater.* **2024**, *34*, 735.
